# Serum Insulin-Like Growth Factor Axis and the Risk of Pancreatic Cancer: Systematic Review and Meta-Analysis

**DOI:** 10.3390/nu9040394

**Published:** 2017-04-18

**Authors:** Yuanfeng Gong, Bingyi Zhang, Yadi Liao, Yunqiang Tang, Cong Mai, Tiejun Chen, Hui Tang

**Affiliations:** 1 Department of Hepatobiliary Surgery, The Affiliated Cancer Hospital& Institute of Guangzhou Medical University, Guangzhou 510095, China; medgongyf@126.com (Y.G.); medliaoyd@126.com (Y.L.); medmaic@126.com (C.M.); medchentj@126.com (T.C.); medtangh@126.com (H.T.); 2 Department of Ultrasound, the First People’s Hospital of Yichang, China Three Gorges University, Yichang 443000, China; yczhangyb@126.com

**Keywords:** insulin-like growth factor, insulin-like growth factor binding protein, pancreatic cancer, morbidity, meta-analysis

## Abstract

Objective: To investigate the association between serum concentration of insulin-like growth factor (IGF) and the risk of pancreatic cancer (PaC). Methods: We identified eligible studies in Medline and EMBASE databases (no reference trials from 2014 to 2016) in addition to the reference lists of original studies and review articles on this topic. A summary of relative risks with 95% confidence intervals (CI) was calculated using a random-effects model. The heterogeneity between studies was assessed using Cochran *Q* and *I*^2^ statistics. Results: Ten studies (seven nested case-control studies and three retrospective case-control studies) were selected as they met our inclusion criteria in this meta-analysis. All these studies were published between 1997 and 2013. The current data suggested that serum concentrations of IGF-I, IGF-II and insulin-like growth factor binding protein-3 (IGFBP-3)in addition to the IGF-I/IGFBP-3 ratio were not associated with an increased risk of PaC (Summary relative risks (SRRs) = 0.92, 95% CI: 0.67–1.16 for IGF-I; SRRs = 0.84, 95% CI: 0.54–1.15 for IGF-II; SRRs = 0.93, 95% CI: 0.69–1.17 for IGFBP-3; SRRs = 0.97, 95% CI: 0.71–1.23 for IGF-I/IGFBP-3 ratio). There was no publication bias in the present meta-analysis. Conclusion: Serum concentrations of IGF-I, IGF-II, IGFBP-1 and IGFBP-3 as well as the IGF-I/IGFBP-3 ratio were not associated with increased risk of PaC.

## 1. Introduction

Pancreatic cancer (PaC) ranks as the fourth most common cause of death from cancer in both men and women in the United States [1]. Despite decades of effort by clinicians and scientists, the five-year survival rate remains poor, as it has only reached a maximum of 5%. The incidence and mortality rates of PaC in the United States have remained stable over the past two decades [2]. However, the incidence rate of PaC in males from China rose in the period from 2000 to 2011 [3]. Radical resection is the only potentially curative therapy. As it is difficult to diagnose in its early stage, approximately 80% of patients cannot receive surgical resection. The five-year survival rate is about 20% after resection. Patients suffering from local, advanced, unresectable or metastatic disease can undergo chemotherapy or chemo-radiotherapy if these patients have a good performance status. A considerable number of epidemiological studies and meta-analyses have investigated possible risk factors of PaC. The association between cigarette smoking and PaC has been demonstrated by nearly all published studies [4,5,6]. Several meta-analyses have suggested that obesity is a risk factor for pancreatic cancer [7,8]. Pancreatitis, especially chronic pancreatitis, was associated with a significantly increased risk of PaC [9], with this risk appearing to be the highest in rare types of pancreatitis, such as hereditary pancreatitis and tropical pancreatitis [10]. New-onset diabetes was associated with a significantly increased risk, meaning it could be a potential clue for the early diagnosis of PaC [11]. Cholelithiasis [12], cholecystectomy [13] and gastrectomy [14] may also increase the risk of PaC.

The insulin-like growth factor (IGF) axis includes two growth factors, IGF-I and IGF-II, in addition to several IGF binding proteins (IGFBP-1 to IGFBP-6), which work together to regulate the amount of free IGF-I and IGF-II in serum. IGFs have long been known as nutritional biomarkers, which are dysregulated in states of under- and over-nutrition. Serum concentration of IGF-I falls rapidly in malnutrition and responds promptly to refeeding, so it may convey the messages of nutritional status, monitoring effect of nutritional support [15].

Recently, there has been growing interest in its role in health and disease, especially in cancers. IGF-I and the IGF-I receptor are highly expressed on the surface of pancreatic cancer cell lines, which initiate intracellular signaling transduction associated with proliferation, invasion and expression of mediators of angiogenesis. Meta-analyses of present studies confirmed previous reports regarding elevated serum levels of IGF-I and IGF-II to be associated with an increased risk of colorectal cancer [16,17], breast cancer [18] and prostate cancer [19]. A high level of IGFBP-3 was associated with a reduced risk of lung cancer [20]. However, the findings have been somewhat contrary to present studies of pancreatic cancer [21,22]. Therefore, we performed this first systematic review and meta-analysis of all available evidence of observational studies, following the meta-analysis of observational studies in epidemiological guidelines to clarify the association between the serum IGF axis and risk of PaC.

## 2. Materials and Methods

### 2.1. Data Sources and Searches

Two authors (Y.G. and Y.L.) independently performed a literature search using Medline and EMBASE databases for articles dated up to 1 May 2016. We searched the studies with the following text words and/or Medical Subject Heading (MeSH) terms: (“IGF” OR “insulin-like growth factor”, “IGFBP” OR “insulin-like growth factor binding protein”) AND (“pancreas”, “cancer” or “adenocarcinoma” or “neoplasm” or “tumor”).

### 2.2. Study Selection

We included studies that met all the following criteria: (1) published as an original article; (2) used a case-control, cross-sectional, nested case-control or cohort design; (3) explored the serum level of IGFs and IGFBPs; (4) studied outcome was incidence of mortality of pancreatic cancer; and (5) estimated odds ratio (OR) or relative risk (RR) with corresponding 95% confidence intervals (CIs) (or data to calculate them) for the highest versus non/lowest levels of insulin-like growth factors and insulin-like growth factor binding proteins were reported. Two authors (Y.G. and Y.L.) independently evaluated all the studies retrieved from the databases. If there were multiple publications from the same study, the most relevant was selected, with other publications used to clarify methodology or characteristics of the population. We did not contact the authors for detailed information of primary studies.

### 2.3. Data Extraction and Quality Assessment

Three authors (C.M., T.C. and H.T.) independently evaluated all the studies retrieved according to the prespecified selection criteria. Any discrepancies between reviewers were addressed by a joint reevaluation of the original article. The following information from each study was extracted using a standardized data collection form: the first author’s last name, year of publication, geographic location, study design, sample size, quality of each study, exposure of interest, concentration levels, the effect estimates with 95% CIs and covariates adjusted in the statistical analysis.

The quality of each study was evaluated independently by three reviewers using the Newcastle-Ottawa Scale (NOS). The NOS consists of three parameters of quality: selection, comparability and outcome (cohort studies) or exposure (case-control studies). The NOS assigns a maximum of four points for selection, a maximum of two points for comparability and a maximum of three points for exposure or outcome. Any discrepancies between reviewers were addressed by a joint reevaluation of the original article.

### 2.4. Statistical Analysis

For simplicity, all measures were interpreted as relative risks (RR) with no distinction between the various estimates (i.e., OR, rate ratio, hazard ratio).As different studies might report different exposure categories (thirds or quarters), we used the study-specific relative risk for the highest versus the lowest category of IGFs and IGFBPs for the meta-analysis. We transformed the corresponding CIs into the log RRs, using the Greenland formula to calculate the corresponding variances. For studies that lacked estimates, we calculated crude estimates from tabular data [23,24,25]. We pooled these relative risks using a fixed effects model to get a summary relative risk for further meta-analysis. We used Woolf’s formula to evaluate the standard error (SE) of the log RRs. Summary relative risks (SRR) with their corresponding 95% CIs were combined and weighted to produce pooled RRs using a fixed- or random-effects model, according to *I*^2^ statistics.

To investigate the sources of heterogeneity across these studies, we carried out heterogeneity tests and sensitivity analyses. In heterogeneity tests, we used the Cochran *Q* and *I*^2^ statistics [26], which were used to test the differences obtained between studies due to chance. For the *Q* statistic, a *p*-value of less than 0.10 was considered representative of statistically significant heterogeneity. The *I*^2^ statistic is the proportion of total variation contributed by variation between studies. It has been suggested that *I*^2^ values of 25%, 50% and 75% are assigned to low, moderate and high heterogeneity, respectively [27]. We conducted the sensitivity analyses to estimate the influence of each individual study on the summary results by repeating the random-effects meta-analysis after omitting one study at a time. We evaluated the role of several potential sources of heterogeneity by sub-group analyses according to adjustments for confounding variables: alcohol consumption, smoking and diabetes mellitus (DM).

Funnel plots and Egger’s test were performed to test evidence of publication bias [28]. Meta-analyses were carried out using STATA12.0 (Stata Corp, College Station, TX, USA).

## 3. Results

### 3.1. Data Sources and Searches

**Figure 1:**
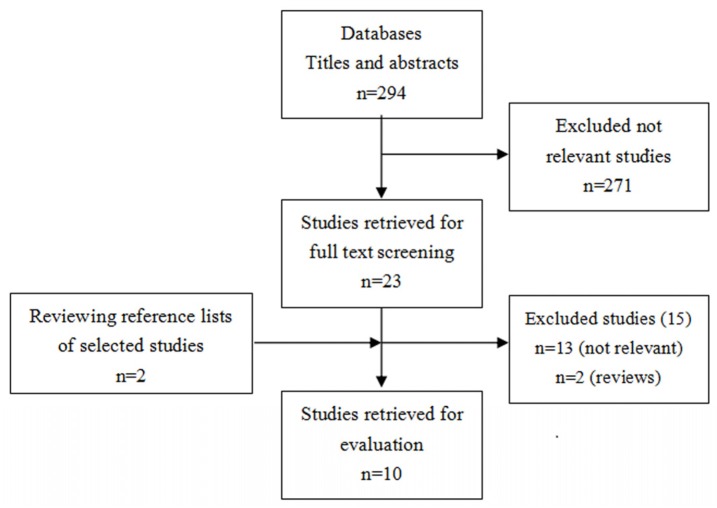
Flow chart of selection of studies included in the meta-analysis.

The detailed steps of our literature search are presented in Figure 1. In brief, a total of 294 citations were obtained for review of the title and abstract. Of the 294 citations, 271 were not relevant. Full texts of the remaining 25 studies were retrieved for review. Thirteen studies were excluded because, when reviewed in detail, their data were not relevant. Two studies were excluded as they were review articles. Finally, 10 studies were included in the final meta-analysis (Figure 1).

### 3.2. Study Characteristics

Ten articles that met our inclusion criteria in this meta-analysis were published between 1997 and 2013 (no reference trials from 2014 to 2016). There were seven nested case-control studies [21,22,29,30,31,32,33,34] and three retrospective case-control studies [23,24,25]. Nine articles described the association between IGF-I concentration and PaC risk [21,22,23,24,25,30,31,32,33], three described the association between IGF-II concentration and PaC risk [22,30,31], six reported the association between IGFBP-3 concentration and PaC risk [21,22,30,31,32,33], while four reported the association between the IGF-I/IGFBP-3 ratio and PaC risk [21,22,30,32]. The average score for the quality assessment of included studies was 7.9 (Table 1).

**Figure 2:**
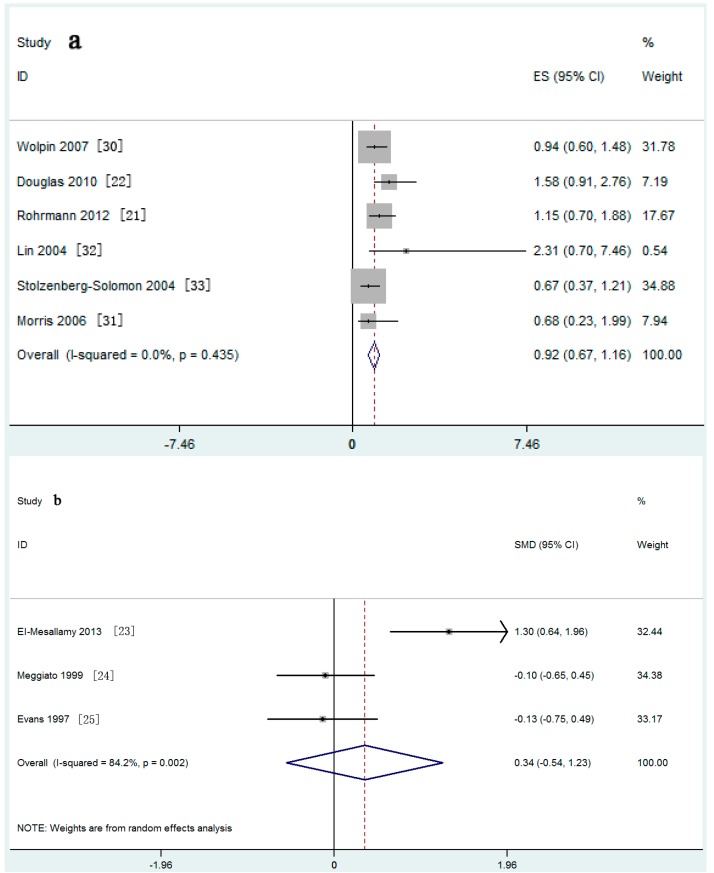
Forest plot of insulin-like growth factor (IGF)-I and pancreatic cancer (PaC) risk for: (**a**) nested case-control studies and (**b**) case-control studies.

**Figure 3:**
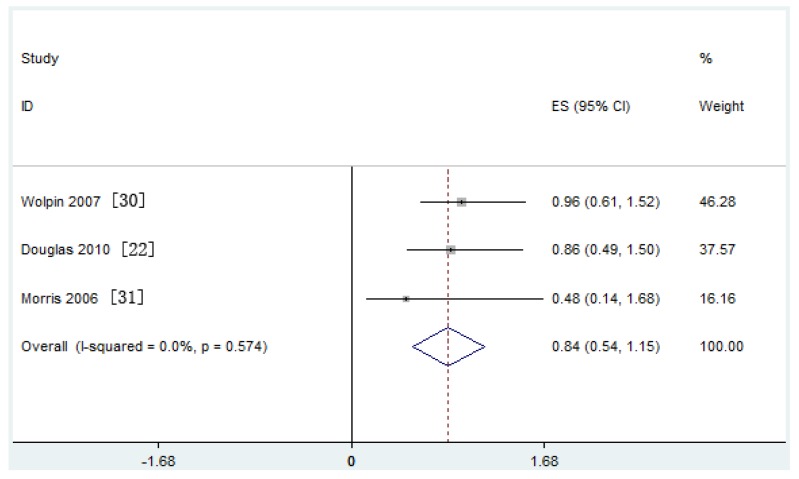
Forest plot of insulin-like growth factor (IGF-II) and pancreatic cancer (PaC) risk. CI = Confidence interval; ES = Effect size.

Meta-analysis of six nested case-control studies in a fixed-effects model found that the serum IGF-I concentration was not associated with the risk of PaC (SRRs = 0.92, 95% CI: 0.67–1.16; test for heterogeneity *p* = 0.435, *I*^2^ = 0.0%). A similar result was found in three retrospective case-control studies (Figure 2a,b). The serum IGF-II concentration was also not related with the risk of PaC (SRRs = 0.84, 95% CI: 0.54–1.15; test for heterogeneity *p* = 0.574, *I*^2^ = 0.0%) (Figure 3).

### 3.3. Meta-Analysis

**Figure 4:**
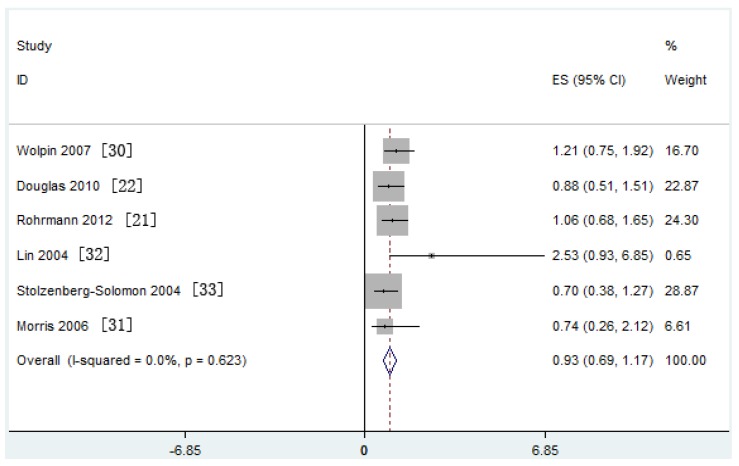
Forest plot of Insulin-like growth factor binding protein (IGFBP)-3 and pancreatic cancer (PaC) risk. CI = Confidence interval; ES = Effect size.

Meta-analysis of six nested case-control studies in a fixed-effects model showed that serum IGFBP-3 concentration was also not associated with the risk of PaC (SRRs = 0.93, 95% CI: 0.69–1.17; test for heterogeneity *p* = 0.623, *I*^2^ = 0.0%) (Figure 4).

**Figure 5:**
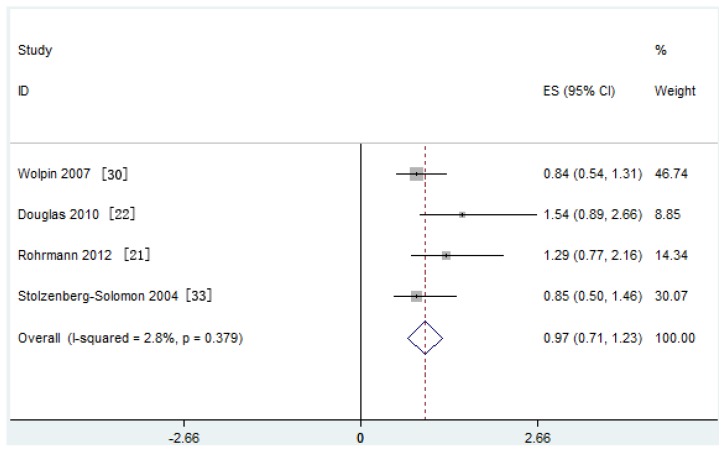
Forest plot of the insulin-like growth factor (IGF)-I/Insulin-like growth factor binding protein (IGFBP)-3 ratio and pancreatic cancer (PaC) risk. CI = Confidence interval; ES = Effect size.

Similarly, meta-analysis of four nested case-control studies in a fixed-effects model showed that the serum IGF-I/IGFBP-3 ratio was not associated with the risk of PaC (SRRs = 0.97, 95% CI: 0.71–1.23; test for heterogeneity *p* = 0.379, *I*^2^ = 2.8%) (Figure 5).

In a sensitivity analysis, the overall homogeneity and effect size was calculated by removing one study at a time. The direction of effect did not change when any study was excluded, supporting the stability of the lack of correlation of IGF-I, IGF-II and IGFBP-3 concentration as well as the IGF-I/IGFBP-3 ratio with an increased risk of PaC.

We subsequently conducted a sub-group systematic review and meta-analysis according to adjustments for confounding variables: alcohol consumption, smoking and DM. Alcohol consumption, smoking and DM are important confounders for risk of PaC. When we limited the meta-analysis to studies that controlled for one of the above confounders or all of them, no positive association was found (Table 2). 

One study reported the association between IGFBP-1 and risk of PaC. IGFBP-1 was not associated with risk of PaC (RR = 0.56, 95% CI: 0.31–1.01) (Table 1).

### 3.4. Publication Bias

**Figure 6:**
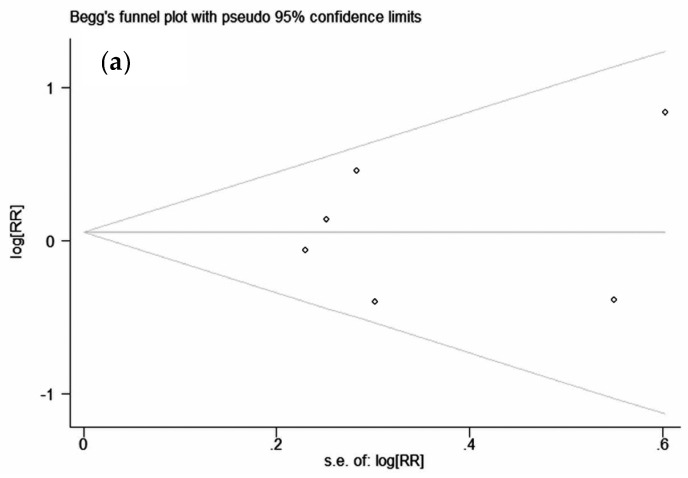

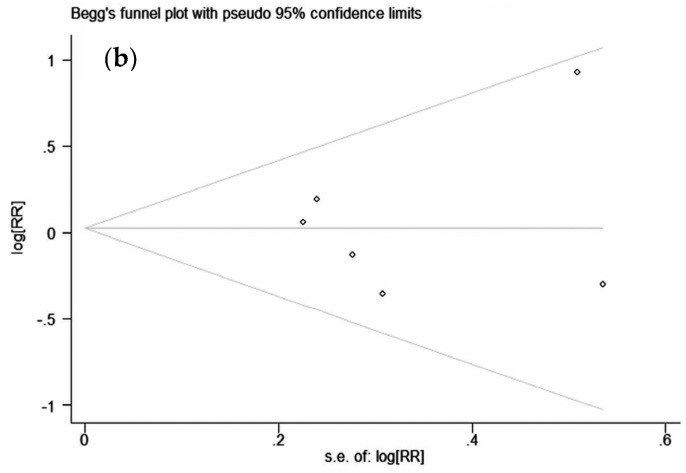
Funnel plot of studies evaluating the association of (**a**) insulin-like growth factor (IGF)-I with pancreatic cancer (PaC) risk (Begg’s test (*p* = 1.00) [21,22,30,31,32,33], Egger’s test (*p* = 0.794) [21,22,30,31,32,33]); and (**b**) IGFBP-3 with PaC risk (Begg’s test (*p* = 0.707) [21,22,30,31,32,33], Egger’s test (*p* = 0.785) [21,22,30,31,32,33]. RR = Relative risk.

The shape of the funnel plots for studies examining the association of IGF-I and IGFBP-3 concentration with PaC risk seemed symmetrical, indicating no publication bias (Figure 6).

## 4. Discussion

In this collaborative meta-analysis, the results showed for the first time that serum IGF-I, IGF-II, IGFBP-1 and IGFBP-3 concentrations as well as the IGF-I/IGFBP-3 ratio were not associated with risk of PaC. Sub-group analysis also did not show any significant associations. The conclusion was contrary to the results found in a meta-analysis of colorectal cancer[16], which showed that IGF-I and IGF-II significantly increased colorectal cancer risk (19 studies included, OR = 1.25, 95% CI: 1.08–1.45 for IGF-I; OR = 1.52, 95% CI: 1.16–2.01 for IGF-II), meta-analysis of breast cancer [17], which showed that IGF-I is positively associated with an increased risk of breast cancer (17 studies included, OR = 1.28, 95% CI: 1.14–1.44), and meta-analysis of lung cancer [35], which showed that IGFBP-3 was inversely associated with an increased risk of this cancer (six studies included, OR = 0.68, 95% CI: 0.48–0.88). 

Interest in IGF-I utilized as a nutritional biomarker began in 1973 when its serum concentration was observed to fall in malnutrition [36]. A previous study showed that starvation, fasting and caloric restriction all resulted in a decrease in serum IGF-I concentration, the physiological function of which was to convert substrates to energy production. Low IGF-I concentration leads to protein catabolism in skeletal muscle, transferring amino acids for hepatic gluconeogenesis, which maintains the glucose level needed to keep the main organs functioning. The decrease in IGF-I concentration also results in enhanced growth hormone (GH) secretion, which enhances hepatic gluconeogenesis by antagonizing insulin’s suppressive function and also by providing more amino acids from muscle [15]. Furthermore, the decrease in IGF-I concentration was more obvious in those with protein and energy malnutrition compared with protein malnutrition alone [37]. However, optimal intakes of both protein and energy are necessary for maintaining an appropriate IGF-I level [38]. The serum IGF-I level appears to be sensitive to both the amount and type of fat provided in nutritional support. Fish oil and low fat formula was significantly related to a faster recovery of the serum IGF-I concentration [39].

IGFs share structural homology and in vitro metabolic activity with insulin, both of which play an important role in proliferation and differentiation of normal and malignant cells. However, they have different receptors. The affinity of the IGF receptor for IGFs is 1000 times greater than that for insulin, while the insulin receptor shows 100 times greater affinity to insulin than that for IGFs [40]. The insulin-like growth factor axis is composed of two ligands (IGF-I and IGF-II), three cell-membrane receptors (insulin receptor (IR), IGF-I receptor (IGF-IR) and IGF-II receptor (IGF-IIR)) and six high-affinity IGF binding proteins (IGFBP-1 to IGFBP-6). Insulin is the main regulator of glucose metabolism, but the IGF axis also exerts insulin-like actions and increases insulin sensitivity. Recombinant human IGF-I could increase insulin sensitivity and improves glycemic control in type 2 diabetes mellitus (T2DM) [41]. The serum concentration of IGF-I was independently associated with insulin sensitivity in subjects with different degrees of glucose tolerance [42]. IGFBPs might influence the risk of DM. The decrease in IGF levels, controlled by the increase of IGFBP-1, served to protect against possible insulin-like activity of the IGFs during fasting [43]. An elevation in IGFBP-1 decreased free IGF-I in serum and muscle protein synthesis under stress conditions [44]. However, the association of the IGF axis with DM might not be causal or pathological. Basic research showed that early T2DM and impaired glucose tolerance are usually characterized by insulin resistance and hyperinsulinemia. Insulin could stimulate hepatic IGF-I synthesis, suppress hepatic IGFBP-1 synthesis in the liver, which could lead to an increase in the serum concentration of IGF-I. Thus, high serum IGF-I levels in patients with T2DM might be due to high insulin levels rather than the biological impact of the IGF axis on DM pathogenesis [45].

The association between IGF axis and risk of PaC is biologically plausible. About 99% of IGFs were combined with IGFBPs. Less than 1% of IGFs were free in serum. Free IGFs in circulation plays an important role in the regulation of cell behavior by binding to its receptor. Nevertheless, IGFBPs can inhibit the activities of IGFs by competitively binding to it and thereby reducing its bioavailability. Furthermore, in vitro experiments showed that exogenously adding insulin, IGF-I and IGF-II stimulated the growth of PaC cell lines via the PI3-kinase pathway, while the mitogenic effects were markedly blocked by providing anti-insulin receptor substrate-1 antibody or PI3-kinase inhibitor [46,47]. Small interfering RNA targeting IGF-IR [48] or anti-IGF-IR antibody [49,50] could be effective and efficient against the growth and metastasis of PaC cell lines. 

Based on the compelling preclinical rationale, the IGF axis showed great promise in the diagnosis of PaC. The combination of the carbohydrate cancer antigen 19-9, IGF-I and albumin resulted in a combined area under the curve of 0.959 with 93.6% sensitivity and 95% specificity, much higher than CA 19-9 alone[51]. However, clinical trials targeting the IGF axis produced disappointment results. A phase II randomized, double-blind, placebo-controlled trial showed that ganitumab (monoclonal antibody inhibitor of IGF-IR, AMG 479) combined with gemcitabine had manageable toxicity but did not improve overall survival [52]. Similarly, adding another IGF-IR inhibitor, cixutumumab to erlotinib and gemcitabine also did not lead to longer progress-free survival or overall survival in metastatic PaC [53]. However, a randomized phase II study showed that higher levels of IGF-I, IGF-II and IGFBP-3 or lower levels of IGFBP-2 were associated with improved overall survival in metastatic PaC patients treated with ganitumab versus placebo [54]. Based on the results of the present study, scientists and clinicians should reconsider the role of the IGF axis in the development and progression of PaC in addition to the targeted therapies focused on the IGF axis.

There are three strengths of the present study. (1) To our knowledge, this study is the first to investigate the association between the IGF axis and risk of PaC; (2) Although limited studies were included, we have performed a comprehensive and systematic search of the literature by using an extensive search strategy; (3) The majority of the included studies were nested case-control studies, which could be effectively controlled for confounding factors.

This meta-analysis has limitations that affect interpretation of the true results. First, all studies in this meta-analysis used a nested case-control study or case-control design, which was more susceptible to recall and selection biases. Second, this investigation did not have sufficient information to perform sub-group analysis, which might affect the stability of the results due to heterogeneity across studies, and might miss some positive results in sub-groups. The carcinogenesis initiated or promoted by trace amounts of growth factors is complicated and naturally lasts for longer periods of time, so follow-up time is one of the most important factors affecting the result. For example, serum transforming growth factor-β1 was not associated with an increase in pancreatic cancer risk. However, this association differed significantly by follow-up time. Higher risk was observed during follow-up time of more than 10 years (OR = 2.13, 95% CI: 1.23–3.68) [55]. Finally, unmeasured or uncontrolled confounding inherited from original studies is a concern in this meta-analysis. Most estimate risks were derived from multivariable models, but individual studies did not adjust for potential confounding factors in a consistent way.

## 5. Conclusions

Our meta-analysis of observational studies provided evidence for the first time that serum IGF-I, IGF-II, IGFBP-1 and IGFBP-3 concentrations as well as the IGF-I/IGFBP-3 ratio were not associated with an increased risk of PaC. Given the small number of studies included in this meta-analysis, limited details and the non-randomized controlled study designs, further prospective cohort studies with a larger sample size and more accurate assessment of baseline characteristics in addition to being well-controlled for confounding factors are needed to affirm the effect of the IGF axis on PaC. 

## Figures and Tables

**Table 1:** Characteristics of the ten included studies.

Author/References	Study Published/Location	Study Design	Cases	Controls	NOS	Exposure of Interest	Concentration Levels	Effect Estimate (95%CI)	Adjustments
Rohrmann [21]	2012/Europe	NCC	422	422	9	IGF-1	Q4 vs. Q1	1.15 (0.70, 1.88)	Age, sex, education, BMI, physical activity, alcohol consumption, smoking and DM
						IGFBP-3		1.06 (0.68, 1.65)	
						IGF-1/IGFBP-3		1.29 (0.77, 2.16)	
Douglas [22]	2010/USA	NCC	187	374	9	IGF-1	Q4 vs. Q1	1.58 (0.91, 2.76)	Age, race, sex, smoking, education, BMI, physical activity, DM, alcohol consumption and nutrients intake
								M: 1.56 (0.78, 3.14)	
								F: 1.74 (0.67, 4.51)	
						IGF-2	Q4 vs. Q1	0.86 (0.49, 1.50)	
								M: 0.75 (0.36, 1.56)	
								F: 0.86 (0.49, 1.50)	
						IGFBP-3	Q4 vs. Q1	0.88 (0.51, 1.51)	
								M: 1.03 (0.51, 2.09)	
								F: 0.77 (0.32, 1.83)	
						IGF-1/IGFBP-3	Q4 vs. Q1	1.54 (0.89, 2.66)	
								M: 1.39 (0.69, 2.80)	
								F: 1.47 (0.58, 3.75)	
Wolpin [30]	2007/USA	NCC	212	635	9	IGF-1	Q4 vs. Q1	0.94 (0.60, 1.48)	Age, sex, smoking, BMI, physical activity, DM, vitamin use and energy intake
						IGF-2		0.96 (0.61, 1.52)	
						IGFBP-3		1.21 (0.75, 1.92)	
						IGF-1/IGFBP-3		0.84 (0.54, 1.31)	
Wolpin [29]	2007/ USA	NCC	144	429	9	IGFBP-1	Q4 vs. Q1	0.56 (0.31,1.01)	Age, sex, smoking, BMI, physical activity, DM, vitamin use and energy intake
Morris [31]	2006/UK	NCC	38	114	8	IGF-1	T3 vs. T1	0.68 (0.23, 1.99)	Age, smoking
						IGF-2		0.48 (0.14, 1.68)	
						IGFBP-3		0.74 (0.26, 2.12)	
Lin [32]	2004/Japan	NCC	69	207	8	IGF-1	Q4 vs. Q1	2.31 (0.70, 7.64)	Age, sex, BMI, smoking and DM
						IGFBP-3		2.53 (0.93, 6.85)	
Stolzenberg-Solomon [33]	2004/Finland	NCC	93	400	8	IGF-1	T3 vs. T1	0.67 (0.37, 1.21)	Age, BMI, smoking, energy intake, alcohol consumption
						IGFBP-3		0.70 (0.38, 1.27)	
						IGF-1/IGFBP-3		0.85 (0.50, 1.46)	
EI-Mesallamy [23]	2013/Egypt	CC	23	20	7	IGF-1 (ug/L)	Continuous	1.30 (0.64, 1.96) ^a^	Age, sex, race
						IGFBP-3 (ug/L)		−1.04 (−1.68, −0.40) ^a^	
						IGF-1/IGFBP-3		1.48 (0.80,2 .16) ^a^	
Meggiato [24]	1999/Italy	CC	35	22	6	IGF-1 (ug/L)	Continuous	−0.10 (−0.65,0.45) ^a^	
Evans [25]	1997/UK	CC	20	20	6	IGF-1 (ug/L)	Continuous	−0.13 (−0.75, 0.49) ^a^	Age
						IGF-2 (U/mL)		−0.50 (−1.13, 0.13) ^a^	
						IGFBP-3 (nmol/L)		−0.20 (−0.82, 0.42) ^a^	

Abbreviations: M = male; F = female; NCC = nested case-control study; CC = case-control study; Q = quartile; T = tertile. ^a^ Mean difference (95%CI). BMI = Body mass index; CI = Confidence interval; DM = Diabetes mellitus; IGF = Insulin-like growth factor; IGFBP = Insulin-like growth factor binding protein.

**Table 2:** Sub-group analysis of relative risks for the association between IGF-I and IGFBP-3 with pancreatic cancer.

Subgroup	References	Relative Risk (95% CI)	Tests for Heterogeneity	
			*I*^2^ (%)	*p*
IGF-I				
Adjustment for alcohol				
Yes	[21,22,33]	0.92 (0.60, 1.24)	48.7	0.142
No	[30,31,32]	0.91 (0.52, 1.30)	0	0.625
Adjustment for DM				
Yes	[21,22,30,32]	1.10 (0.77, 1.43)	0	0.560
No	[31,33]	0.67 (0.29, 1.05)	0	0.984
Adjustment for smoking, alcohol and DM				
Yes	[21,22]	1.27 (0.78, 1.77)	0	0.442
No	[30,31,32,33]	0.80 (0.51, 1.08)	0	0.661
IGFBP-3				
Adjustment for alcohol				
Yes	[21,22,33]	0.87 (0.59, 1.14)	0	0.562
No	[30,31,32]	1.12 (0.63, 1.60)	0	0.448
Adjustment for DM				
Yes	[21,22,30,32]	1.05 (0.75, 1.35)	0	0.638
No	[31,33]	0.71 (0.31, 1.11)	0	0.939
Adjustment for smoking, alcohol and DM				
Yes	[21]	0.97 (0.62, 1.32)	0	0.613
No	[30,31,32,33]	0.89 (0.56, 1.22)	4.1	0.372

CI = Confidence interval; *I*^2^ = *I*-square; DM = Diabetes mellitus; IGF = Insulin-like growth factor.

## Abbreviations

BMI	Body mass index
CI	Confidence interval
DM	Diabetes mellitus
IGF	Insulin-like growth factor
IGFBP	Insulin-like growth factor binding protein
OR	Odds ratio
PaC	Pancreatic cancer
RR	Relative risk
SRRs	Summary relative risks
